# Impaired Neural Differentiation of Induced Pluripotent Stem Cells Generated from a Mouse Model of Sandhoff Disease

**DOI:** 10.1371/journal.pone.0055856

**Published:** 2013-01-31

**Authors:** Yasuhiro Ogawa, Makoto Tanaka, Miho Tanabe, Toshihiro Suzuki, Tadayasu Togawa, Tomoko Fukushige, Takuro Kanekura, Hitoshi Sakuraba, Kazuhiko Oishi

**Affiliations:** 1 Department of Pharmacology, Meiji Pharmaceutical University, Tokyo, Japan; 2 Department of Analytical Biochemistry, Meiji Pharmaceutical University, Tokyo, Japan; 3 Department of Functional Bioanalysis, Meiji Pharmaceutical University, Tokyo, Japan; 4 Department of Dermatology, Kagoshima University Graduate School of Medical and Dental Sciences, Kagoshima, Japan; 5 Department of Clinical Genetics, Meiji Pharmaceutical University, Tokyo, Japan; Massachusetts General Hospital, United States of America

## Abstract

Sandhoff disease (SD) is a glycosphingolipid storage disease that arises from mutations in the *Hexb* gene and the resultant deficiency in β-hexosaminidase activity. This deficiency results in aberrant lysosomal accumulation of the ganglioside GM2 and related glycolipids, and progressive deterioration of the central nervous system. Dysfunctional glycolipid storage causes severe neurodegeneration through a poorly understood pathogenic mechanism. Induced pluripotent stem cell (iPSC) technology offers new opportunities for both elucidation of the pathogenesis of diseases and the development of stem cell-based therapies. Here, we report the generation of disease-specific iPSCs from a mouse model of SD. These mouse model-derived iPSCs (SD-iPSCs) exhibited pluripotent stem cell properties and significant accumulation of GM2 ganglioside. In lineage-directed differentiation studies using the stromal cell-derived inducing activity method, SD-iPSCs showed an impaired ability to differentiate into early stage neural precursors. Moreover, fewer neurons differentiated from neural precursors in SD-iPSCs than in the case of the wild type. Recovery of the *Hexb* gene in SD-iPSCs improved this impairment of neuronal differentiation. These results provide new insights as to understanding the complex pathogenic mechanisms of SD.

## Introduction

Sandhoff disease (SD) is a glycosphingolipid storage disease caused by a deficiency in β-hexosaminidase activity. This deficiency causes aberrant lysosomal accumulation of the ganglioside GM2 and related glycolipids mainly in neuronal cells. Such dysfunctional glycolipid storage causes severe neurodegeneration through a poorly understood pathogenic mechanism. β-hexosaminidase has two major isoforms, namely β-hexosaminidase A (HexA; αβ heterodimer) and β-hexosaminidase B (HexB; ββ homodimer), and a minor isoform, β-hexosaminidase S (HexS; αα homodimer). Human *Hexa* and *Hexb* genes encode α- and β-subunits, respectively. A mutation in *Hexb* causes SD due to deficient activity of HexA and HexB. Because only HexA can degrade GM2 ganglioside, the loss of HexA activity in the brains of SD patients causes progressive GM2 ganglioside accumulation.

Previous studies have shown that the *Hexb-*null (*Hexb*−/−) mouse develops an SD-like illness and therefore provides a useful animal model to investigate the pathophysiology of SD [1]–[3]. As with many other lysosomal storage diseases, neurodegeneration is a prominent feature of SD. Because neurons accumulate a large amount of GM2 ganglioside, relative to other tissues in this disease, it is generally thought that the nervous system is the main pathological target. The accumulation of GM2 ganglioside and its derivatives in the nervous system is implicated in loss of function and aberrant neuronal cell death [4]. However, recent studies have provided strong evidence that shows GM2 ganglioside accumulation cannot account for all of the nervous system damage in *Hexb*−/− mice [5]–[7].

Induced pluripotent stem cells (iPSCs) are prepared by transducing several genes into somatic cells. Their features are similar to those of embryonic stem (ES) cells, and it is possible for iPSCs to differentiate into various cell types. Therefore, iPSCs may replace ES cells as a cell resource that can be applied for regenerative therapy. On the other hand, iPSCs may be useful to clarify the pathogenesis of early embryonic disorders. If disease-specific iPSCs are prepared using cells from patients and compared with normal iPSCs, the etiologies of refractory diseases may be clarified. In this study, we have generated iPSCs from a *Hexb*−/mouse using non-integrating episomal vectors. We also investigated the relationship between β-hexosaminidase deficiency and abnormal neurogenesis *in vitro*.

## Methods

### Mouse Models

*Hexb*−/− mice (C57BL/6×129 sv) [2] were kindly provided by Dr. Richard L. Proia (Genetics of Department and Disease Branch, National Institute of Diabetes, and Digestive and Kidney Disease, National Institutes of Health, Bethesda, MD).

### Isolation and Propagation of SD-Neural Stem Cells

All animal procedures were in accordance with the Guidelines for Animal Experimentation of the Japanese Association for Laboratory Animal Science, and approved by the Institutional Animal Use and Care Committee of Meiji Pharmaceutical University. Neural stem cell (NSC) cultures were performed as described elsewhere with minor modifications [8]. Briefly, cortices from SD mouse embryos on embryonic day 12.5 were triturated in N2 medium (DMEM/F12 plus N2 supplement) containing 20 ng/ml basic fibroblast growth factor (bFGF; R&D Systems Inc., Minneapolis, MN) and 2 µg/ml heparin (Sigma-Aldrich, Tokyo, Japan). Single cells were plated at 1×10^5^ cells/ml in N2 medium. The number of generated primary neurospheres was determined at 5–7 days after plating. Neurospheres were collected by centrifugation and dissociated mechanically in single-cell suspensions. SD-NSCs were then replated in fresh medium. At 3–4 days after plating, neurospheres reformed and could be passaged further. Passage 5 cultures were used for experimentation.

### Generation of iPSCs

PB-TET-MKOS (plasmid 20959; Addgene, Cambridge, MA) was modified by first replacing the CMV promoter with a CAG promoter. To generate an oriP/EBNA1-based episomal construct (LZRS-CX-MKOS), the expression cassette comprising the MKOS sequence (c-Myc, Klf4, Oct4 and Sox2 ORFs linked to 2A peptide sequences) from PB-TET-MKOS was ligated into the BamHI/NotI site in the LZRS retroviral vector backbone [9].

SD-NSCs were seeded onto poly-L-ornithine/fibronectin-coated 35-mm culture dishes at a density of 5×10^4^ cells per dish, and then cultured as monolayers in N2 medium containing B27 supplement minus vitamin A (Invitrogen, Carlsbad, CA), 20 ng/ml bFGF and 20 ng/ml epidermal growth factor (EGF; R&D Systems, Inc.). Reprogramming of SD-NSCs with the LZRS-CX-MKOS plasmid was carried out as described elsewhere [10]. Briefly, episomal plasmids were transfected into SD-NSCs with Lipofectamine 2000 (Invitrogen). From day 6, transfected NSCs were harvested with 0.25% trypsin-EDTA (Invitrogen) and seeded onto 100-mm culture dishes with SNL feeder cells (Riken Cell Bank, Tsukuba, Japan). Colonies with a morphology similar to that of wild-type (WT)-iPSC colonies were readily visible on day 12 post-transfection. On days 25–30, colonies were picked up for expansion on mouse embryonic fibroblast (MEF) feeder cells in mouse ES cell medium containing 1000 U/ml leukemia inhibitory factor (LIF; ESGRO, Chemicon International Inc., Temecula, CA). Medium was changed every other day. Colonies with a morphology similar to that of WT-iPSC colonies were readily visible on day 8 post-transfection. On days 12–16, colonies were picked up for expansion on MEF feeder cells in mouse ES cell medium containing LIF.

### Cell Culture

iPSCs were maintained on mitomycin C-treated MEFs in DMEM (Wako Co., Tokyo, Japan) containing 15% FBS (Invitrogen), 2 mM L-glutamine, 0.1 mM nonessential amino acids, 0.1 mM 2-mercaptoethanol, 50 U/ml penicillin and 50 µg/ml streptomycin as described elsewhere [11]. Mouse ES cells (EB3) were maintained on gelatin-coated dishes in G-MEM (Wako) containing 1% FBS, 10% KSR (Invitrogen), 2 mM L-glutamine, 0.1 mM nonessential amino acids, 1 mM pyruvate, 0.1 mM 2-mercaptoethanol, and 1000 U/ml LIF as described elsewhere [12]. WT-iPSCs (iPS-MEF-Ng-20D-17), EB3 cells and PA6 cells were purchased from the Riken Cell Bank.

### Proliferation Assay

iPSCs were seeded on mitomycin C-treated MEFs at a density of 1.5×10^4^ cells/cm^2^ in 96-well plates, and incubated for up to 53 h. Assays were performed by adding WST-1 (Roche, Tokyo, Japan) directly to the culture wells, followed by incubation for 4 h at 37°C. Plates were then read by a microplate absorbance reader (Model 680; Bio-Rad, Tokyo, Japan) by measuring the absorbance of the dye at a wavelength of 450 nm and a reference wavelength of 655 nm. The net absorbance was obtained by subtracting the value of MEFs alone from that of each sample. Four independent experiments were performed for each experimental condition.

### Immunostaining and Alkaline Phosphatase Staining

Immunostaining was performed as described elsewhere [13]. Briefly, cells were fixed with 4% paraformaldehyde in PBS for 15 min at 4°C, and then incubated in blocking buffer (0.1% Triton X-100 and 10% normal goat serum in PBS). Primary antibodies diluted in blocking buffer were added to cells at appropriate concentrations, incubated at room temperature overnight, and then washed with PBS. Secondary antibodies were incubated at room temperature for 2 h and then washed with PBS. Fluorescence images were obtained under a confocal laser-scanning microscope (FluoView 500; Olympus, Tokyo, Japan) or an AxioImager using an AxioCam MRm digital camera (Carl Zeiss, Tokyo, Japan). AxioVision (Carl Zeiss) acquisition software was used for obtaining images. In some images, the brightness level was subsequently adjusted using Photoshop (Adobe Systems Japan, Tokyo, Japan). No other processing of images was performed. Alkaline phosphatase (AP) activity was detected using a BCIP/NBT Alkaline Phosphatase Substrate Kit (Vector Labs, Burlingame, CA), that stained AP-positive cells as blue.

### Antibodies

A mouse monoclonal antibody against GM2 ganglioside (GMB28; immunoglobulin M) was kindly donated by Dr. Tadashi Tai (Department of Tumor Immunity, The Tokyo Metropolitan Institute of Medical Science, Tokyo, Japan). Anti-SSEA1 (MC-480; Developmental Studies Hybridoma Bank, DSHB, Iowa City, IA), anti-nestin (Rat-401; DSHB), anti-α-smooth muscle actin (Progen, Heidelberg, Germany), anti-neuronal class III β-tubulin (Tuj1; Covance, Richmond, CA), anti-Sox2 (Millipore, Bedford, MA), anti-glial fibrillary acidic protein (GFAP; Dako, Carpenteria, CA), anti-HA.11 (16B12; Covance, Denver, PA), anti-cardiac troponin I (AB Biotec, Stockholm, Sweden), anti-α-fetoprotein (R&D Systems), and anti-cleaved caspase-3 (Asp175; Cell Signaling Technology, Danvers, MA) were used as primary antibodies. The secondary antibodies used were Alexa Fluor 568-conjugated goat anti-mouse IgG, Alexa Fluor 488-conjugated goat anti-mouse IgG_1_, Alexa Fluor 568-conjugated goat anti-mouse IgM, and Alexa Fluor 488-conjugated goat anti-rabbit IgG (all purchased from Molecular Probes, Eugene, OR).

### Spontaneous Differentiation

Undifferentiated iPSCs on feeder cells were trypsinized, and cell suspensions were plated onto tissue culture dishes and incubated for 1 h at 37°C to allow feeder cells to attach. Unattached iPSCs were collected, dissociated into single cells in 0.25% trypsin-EDTA, and then quickly reaggregated in ES cell medium (3000 cells/150 µl/well) using 96-well low cell adhesion plates (Sumilon Spheroid Plates; Bakelite Co., Tokyo, Japan) to initiate embryoid body (EB) formation. After 7 days, cell aggregates in suspension cultures were plated onto gelatin-coated dishes in differentiation medium (DMEM containing 10% FBS, 4 mM L-glutamine, 0.1 mM nonessential amino acid, and 0.1 mM 2-mercaptoethanol). After 10 days, cells were stained with antibodies against cell type-specific markers.

### Induction of Neural Differentiation

For differentiation using the stromal cell-derived inducing activity method (SDIA), SD-iPSCs were cocultured on PA6 stromal cells as single cells at 5000 cells/60-mm dish to form colonies in G-MEM medium containing 10% KSR, 2 mM glutamine, 1 mM pyruvate, 0.1 mM nonessential amino acids, and 0.1 mM 2-mercaptoethanol, and then cultured for 7 days at 37°C with 5% CO_2_, as described elsewhere [12]. To induce primary neurospheres, iPSC colonies were detached by trypsinization, mechanically dissociated into single cells, and then cultured for several days in N2 medium containing 2% B27 supplement minus vitamin A, 20 ng/mL bFGF, and 20 ng/mL EGF.

### Teratoma Formation

Approximately 5×10^5^ cells suspended in a 100 µl Matrigel-GFR (BD Biosciences, San Jose, CA)/medium mixture were injected subcutaneously into the dorsal flanks of nude mice (Balb/c slc−nu/nu, Foxn1−/−; Nihon SLC, Shizuoka, Japan). Mice were sacrificed at 3–5 weeks post-injection, and the injection sites were dissected, fixed with 4% paraformaldehyde in phosphate buffer, and then embedded in Tissue-Tec OCT compound (Sakura Finetek USA Inc., Torrance, CA). Subsequently, 25 µm sections were analyzed by immunostaining.

### RT-PCR

Total RNA was isolated from 5×10^5^ cells using an Isogen RNA purification kit (Nippon Gene, Tokyo, Japan). One microgram of total RNA was reverse transcribed using SuperScript II enzyme (Life Tech., Inc., MD) and oligo (dT)12–18 primers. PCR was performed in 25 µl reaction mixtures as described elsewhere [13]. Primers for Nat1 were used as internal controls to confirm that the amount of input RNA was the same for each sample. The primers used are listed in Table S1 [14], [15]. PCR products were resolved in 1.5% agarose gels containing 0.5 µg/ml ethidium bromide, visualized by ultraviolet transillumination and photographed.

### Electron Microscopy

Cells were detached after selected treatments, pelleted and fixed in 2.5% glutaraldehyde and 2% paraformaldehyde in 0.1 M phosphate buffer, pH 7.4 for 2 h at 4°C, and then post-fixed in 2% osmium tetroxide for 1 h at 4°C. Samples were dehydrated, embedded in Epon 812 resin, and then cut into 70 nm sections for microscopy. Sections were then post-stained with 2% uranyl acetate and Reynolds lead citrate. Samples were viewed under a Hitachi H-7100 electron microscope (Hitachi, Ibaraki, Japan).

### Cardiomyocyte and Neural Stem Cell Differentiation

Lineage-directed differentiation of iPSCs into cardiomyocytes and NSCs was induced by EB formation in suspension cultures, and then attachment cultures.

### TLC Immunostaining

TLC immunostaining was performed as described elsewhere [16]. Spots were visualized with 50% sulfuric acid and evaluated by densitometry using NIH Image software. The GM2 ganglioside concentrations (expressed as ng GM2 per 1×10^6^ cells) were calculated from the TLC data.

### *In vitro* Electroporation

To construct an oriP/EBNA1-based episomal plasmid (pEB-HexB-HA-Neo), HA-tagged HexB (MGC collection #100015010, Invitrogen) generated by PCR with a 5′ primer containing a SalI site and a 3′ primer containing a NotI site was ligated into a XhoI/NotI site of pEBMulti-Neo (Wako). The pEB-HexB-HA-Neo plasmid was electroporated using a pulse generator, Cuy21Pro-vitro (NEPA Gene, Chiba, Japan), following the manufacturer's protocol. Transfected cells were selected with 500 µg/mL G418.

## Results

### Generation of iPSCs from the SD Mouse Models

**Figure 1:**
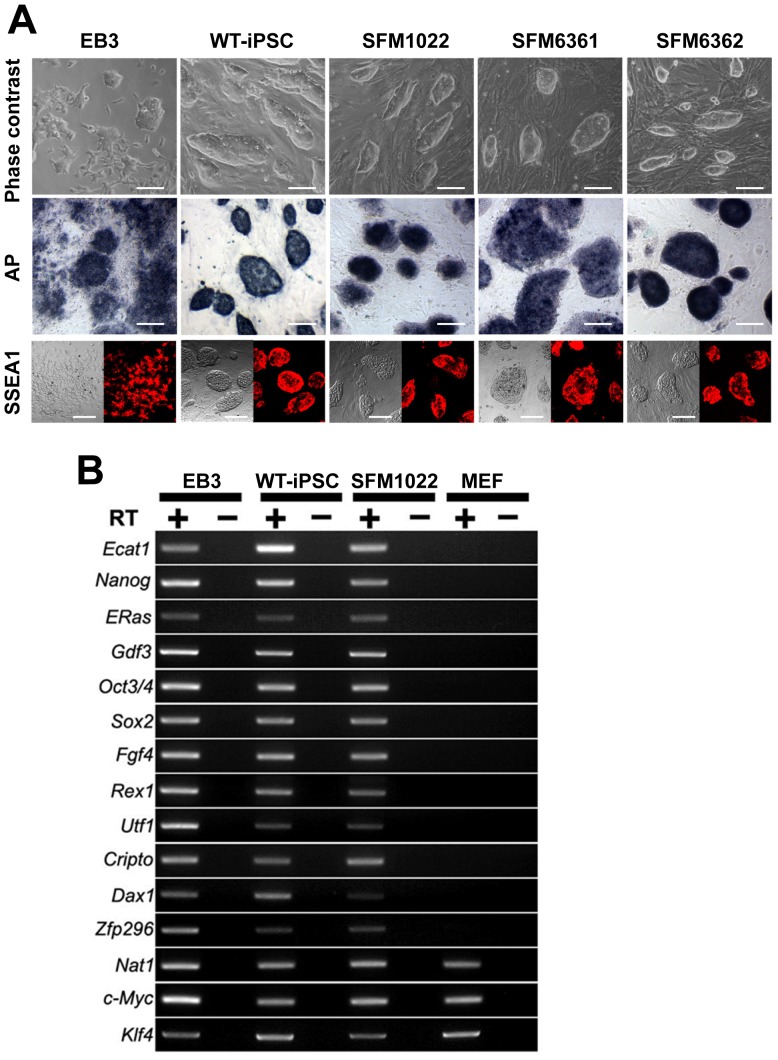
Characterization of iPSCs derived from the NSCs of *Hexb*–/− mice. A, Phase-contrast image, AP staining and SSEA1 immunostaining (left: phase-contrast; right: immunostaining) of SD-iPSC clones (SFM1022, SFM6361, and SFM6362) grown on MEF feeder cells. EB3 (mouse ES cells) and WT-iPSCs were positive controls. Scale bar indicates 100 µm. B, RT-PCR analysis of ES cell marker gene expression in SD-iPSC clone SFM1022, WT-iPSCs, ES cells, and MEFs. Nat1 was used as an internal control. PCR products were amplified from cDNA samples with (+) or without (−) reverse transcriptase.

Transgenes encoding Klf4, Oct3/4, Sox2, and c-Myc were introduced into NSCs from the *Hexb*−/− mice by EBNA1/oriP-based episomal vectors. Four weeks after transfection, colonies were picked up based on their morphological resemblance to mouse WT-iPSCs. Three SD clones (SFM1022, SFM6361, and SFM6362) propagated robustly as colonies when maintained on MEFs (Fig. 1A). The growth rates of the three clones were comparable with that of WT-iPSCs (Fig. S1). To determine whether or not the clones expressed pluripotency markers, we verified the presence of AP activity, as well as immunostained for SSEA1. These colonies were positive for both AP activity and SSEA1. RT-PCR analysis showed that the putative SD-iPSC clone SFM1022 was positive for all 12 ES cell marker genes including Ecat1, Nanog, ERas, Gdf3, Oct3/4, Sox2, Fgf4, Rex1, Utf1, Cripto, Dax1, and Zfp296 (Fig. 1B). Expression of these genes in SFM1022 was similar to that in mouse ES cells and WT-iPSCs, but was absent in MEFs. As previously described, c-Myc and Klf4 are expressed in MEFs, ES cells and WT-iPSCs [17].

**Figure 2:**
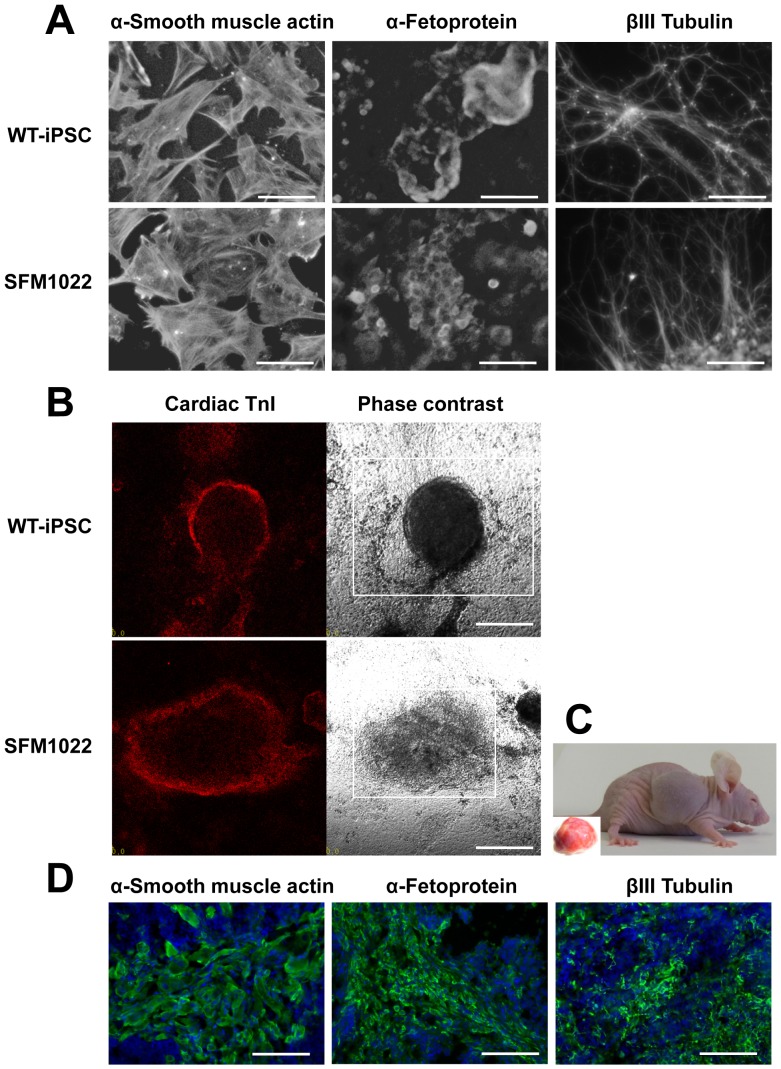
Differentiation of SFM1022 into the cell types of the three germ layers. A, Immunostaining showing that markers for the three germ layers (α-smooth muscle actin, α-fetoprotein, and βIII tubulin) were expressed in spontaneously differentiated SFM1022 cells. WT-iPSCs were used as a positive control. Scale bar indicates 100 µm. B, Differentiation of SFM1022 cells into cardiomyocytes. Between 2 and 5 days after attachment of EBs to gelatin-coated dishes, spontaneously beating areas were observed (Movies S1 and S2). Immunostaining for a cardiomyocyte marker, cardiac troponin I (TnI; red), in beating cell clusters (left: immunostaining; right: phase-contrast). Squares in phase-contrast photographs indicate beating areas. Scale bar indicates 200 µm. C, Teratoma formation of SFM1022 cells after transplantation into nude mice. The inset shows a removed tumor. D, Immunostaining (green) of tumors for α-smooth muscle actin, α-fetoprotein, and βIII tubulin. Blue represents DAPI staining. Scale bar indicates 100 µm.

To determine whether or not SD-iPSC clones differentiated into the cell types of different germ layers, SD-iPSC clones were allowed to spontaneously differentiate in EB cultures. EB formation was achieved by culturing SD-iPSCs in differentiation medium on low-attachment plates for 7 days, followed by transfer to gelatin-coated dishes for further cultures. After 10 days, immunostaining showed that cells were positive for α-smooth muscle actin (mesoderm marker), α-fetoprotein (endoderm marker), and βIII tubulin (ectoderm marker) (Figs. 2A and S2). Between 2 and 5 days after attachment of the EBs to gelatin-coated dishes, spontaneously beating areas were observed (Movies S1 and S2). Immunostaining revealed that the beating cells were positive for the cardiomyocyte marker, cardiac troponin I (Fig. 2B). Next, we verified the pluripotency of SFM1022 in teratoma formation assays. After injection of SFM1022 cells into immunocompromised mice, SFM1022 cells formed teratomas containing tissue derivatives of endoderm, mesoderm, and ectoderm (Fig. 2C, 2D). We conclude that SD-iPSC clones can spontaneously differentiate into derivatives of all three germ layers.

### SD-iPSCs can Differentiate Toward the Neural Lineages

**Figure 3:**
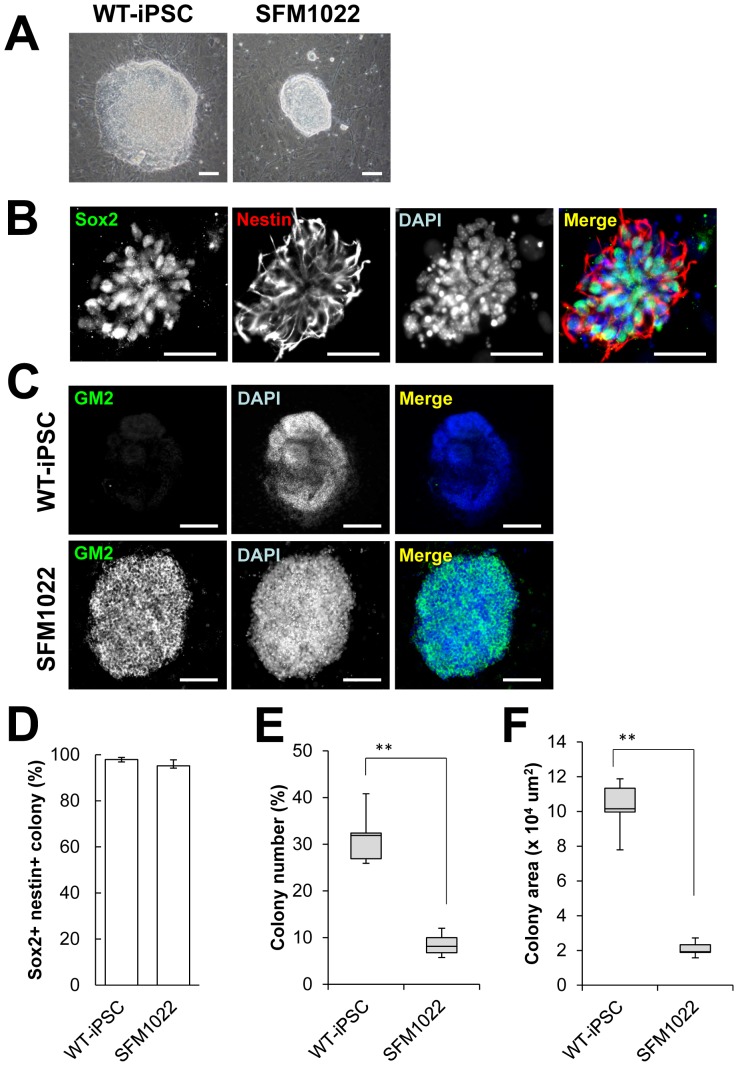
Characterization of SDIA-induced colonies at day 7 of differentiation. A, Phase-contrast image of SFM1022 and WT-iPSC colonies. B, Immunostaining for Sox2 (green) and nestin (red). C, Immunostaining for GM2 (green). Blue represents DAPI staining. Scale bar indicates 100 µm (A) and 500 µm (B, C). D, The percentages of SFM1022 and WT-iPSC colonies that expressed both Sox2 and nestin were determined. Data are the means ± S.E., and were obtained for five independent experiments. The number (as a function of the initial cell number plated) (E) and sizes (F) of SDIA-induced colonies of larger than 100 µm at day 7 of differentiation were determined. Data were analyzed using the Mann–Whitney U test and are shown as box-and-whisker plots. Boxes, 75^th^ percentile with the median indicated; bars, 10^th^ and 90^th^ percentiles. **P<0.01. Data were obtained for five independent experiments.

Lineage-directed differentiation of iPSCs into disease-relevant cell types *in vitro* is important for both mechanistic and therapeutic studies. Because neural cells are one of the major cell types affected by SD, we attempted to induce differentiation toward the neural lineages. SD-iPSCs were cocultured with PA6 stromal cells, as an inducer source. After 7 days, undifferentiated SD-iPSCs formed differentiating colonies containing neural cell lineages (Fig. 3A). Immunostaining of SFM1022-derived colonies revealed the expression of nestin, a neural precursor marker, and Sox2, a marker for neuroepithelial cells and NSCs, indicating the induction of NSCs/precursors (Fig. 3B). The percentages of colonies derived from WT-iPSCs and SFM1022, which expressed both Sox2 and nestin were 97.9±0.9% and 95.2±2.6% (mean±S.E, n = 5), respectively (Fig. 3D). These results indicate that SD-iPSCs cocultured with PA6 cells for 7 days transit through a neural precursor stage. It is noteworthy that, even though the same number of iPSCs was plated on PA6 stromal cells, significantly fewer colonies were formed by SD-iPSCs, compared with those by WT-iPSCs (Figs. 3E and S3A). Moreover, the colonies formed by SD-iPSCs were smaller than those formed by WT-iPSCs (Figs. 3F and S3B).

**Figure 4:**
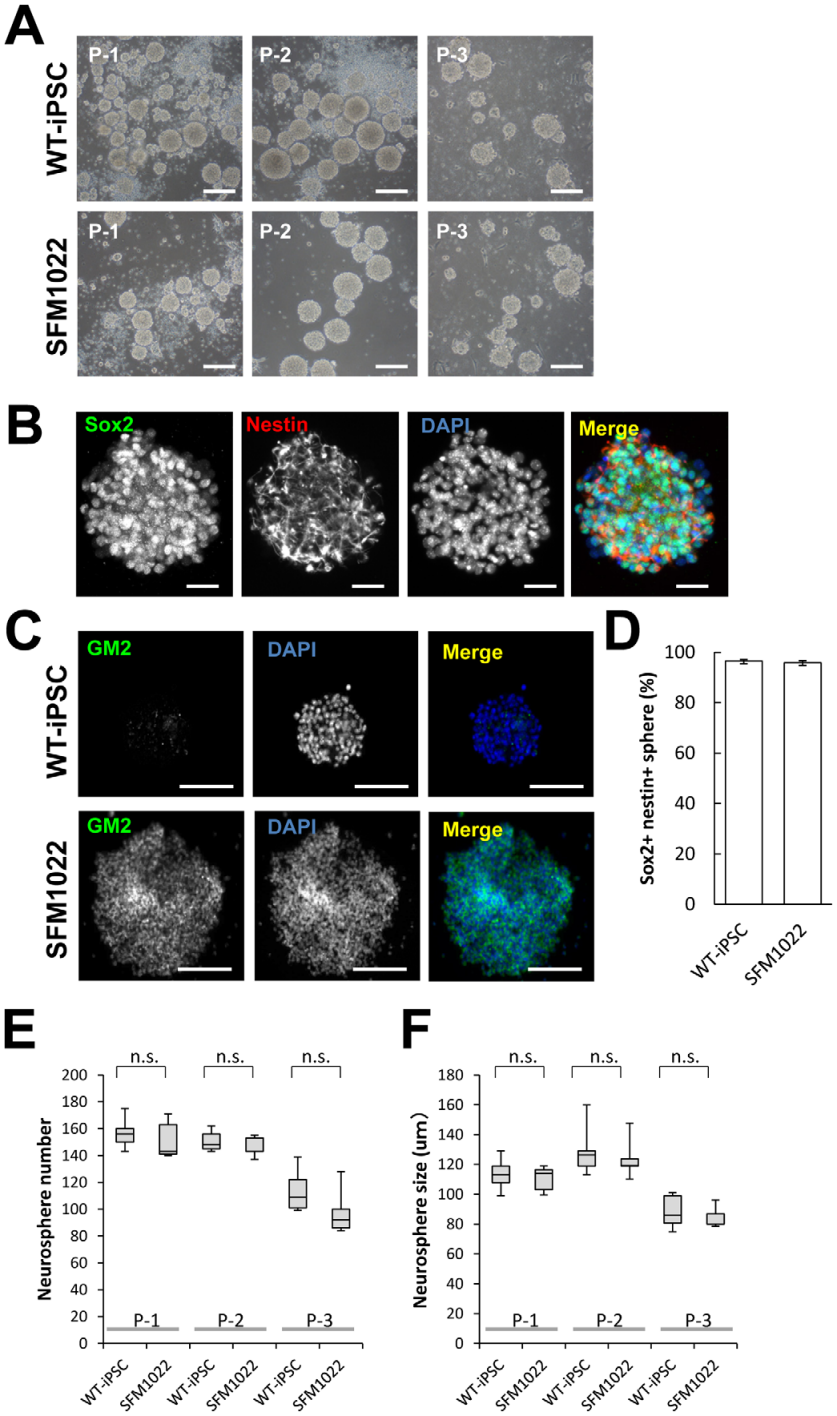
Characterization of neurospheres formed by SDIA-treated colonies. A, Phase-contrast image of passage 1–3 neurospheres formed by SFM1022 cells and WT-iPSCs. B, Immunostaining for Sox2 (green) and nestin (red). C, Immunostaining for GM2 (green). Blue represents DAPI staining. Scale bar indicates 200 µm (A), 50 µm (B) and 20 µm (C). D, The percentages of neurospheres formed by WT-iPSCs and SFM1022 cells, which expressed both Sox2 and nestin were determined. Data are the means ± S.E. and were obtained for five independent experiments. The number (E) and sizes (F) of passage 3–4 neurospheres of larger than 100 µm were determined. Data were analyzed using the Mann–Whitney U test and are shown as box-and-whisker plots. Boxes, 75^th^ percentile with the median indicated; bars, 10^th^ and 90^th^ percentiles. Data were obtained for five independent experiments. n.s.: Difference not significant (*P*>0.05).

To determine whether or not neurospheres derived from SDIA-colonies had the properties of NSCs, colonies were detached by trypsinization, mechanically dissociated into single cells, and then cultured in N2 medium containing 2% B27 supplement minus vitamin A, 20 ng/mL bFGF, and 20 ng/mL EGF. At 2–3 days after dissociation, cells formed a number of spheres. After 3–4 days in culture, the resulting primary spheres were passaged to further increase the number of NSCs. Repeated passaging could be achieved until passage 3 (Fig. 4A). Immunostaining of passage 1 spheres revealed the expression of both Sox2 and nestin, indicating that SDIA-treated iPSCs formed neurospheres (Fig. 4B). The percentages of neurospheres derived from WT-iPSCs and SFM1022, which expressed both Sox2 and nestin were 96.5±0.6% and 95.8±0.9% (mean±S.E., n = 5), respectively (Fig. 4D). There were no differences in the number or size of neurospheres formed by SFM1022 and WT-iPSCs (Fig. 4E, 4F). These results indicate no difference in the ability to form neurospheres between SD-iPSCs and WT-iPSCs.

### Disease Phenotypes of iPSCs

**Figure 5:**
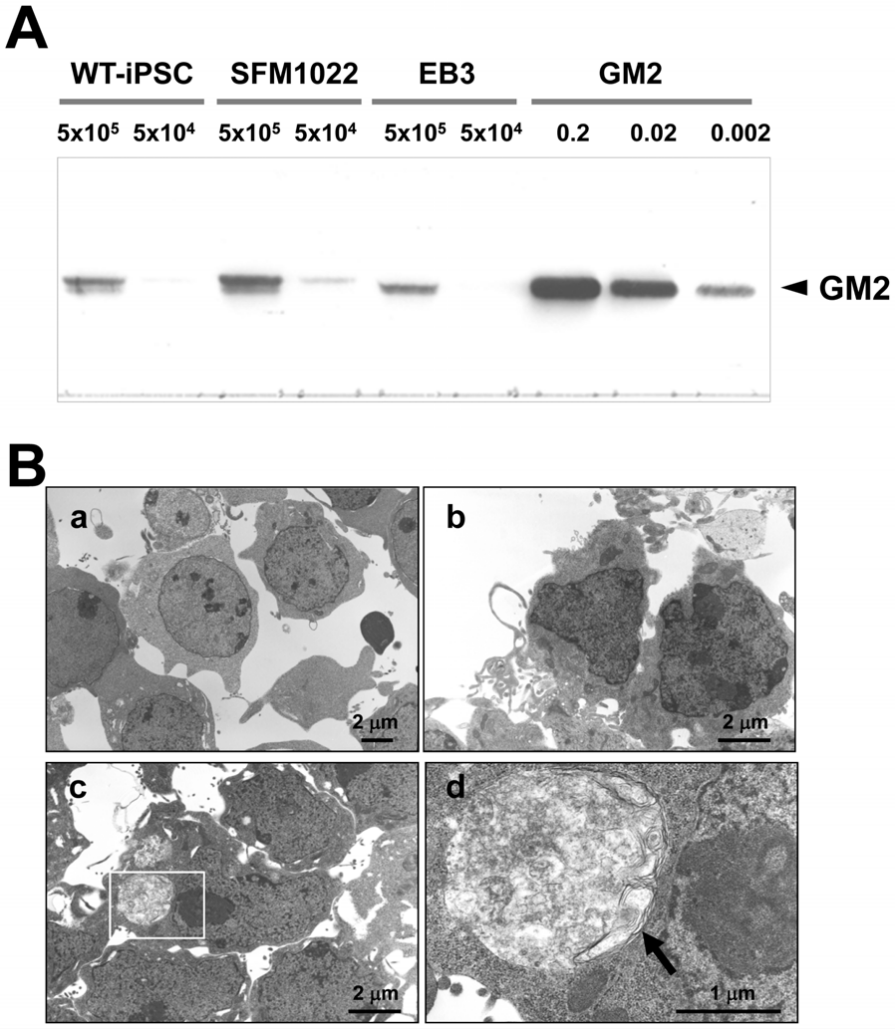
Disease phenotypes of iPSCs. A, TLC immunostaining, with anti-GM2 antibodies, of SFM1022, EB3 cells and WT-iPSCs. Authentic GM2 ganglioside (0.002, 0.02 and 0.2 µg) was used as a control. B, Electron micrographs of differentiated neural cells derived from EB3 cells ( a), WT-iPSCs (b), and SFM1022 cells (c, d). The boxed region in c is magnified in d. Inclusion bodies with a membranous and pleomorphic shape (arrow) were identified in the cytoplasm of SFM1022 cells, but not in that of EB3 cells and WT-iPSCs (a: ×3000, b: ×5000, c: ×5000, d: ×20000).

**Figure 6:**
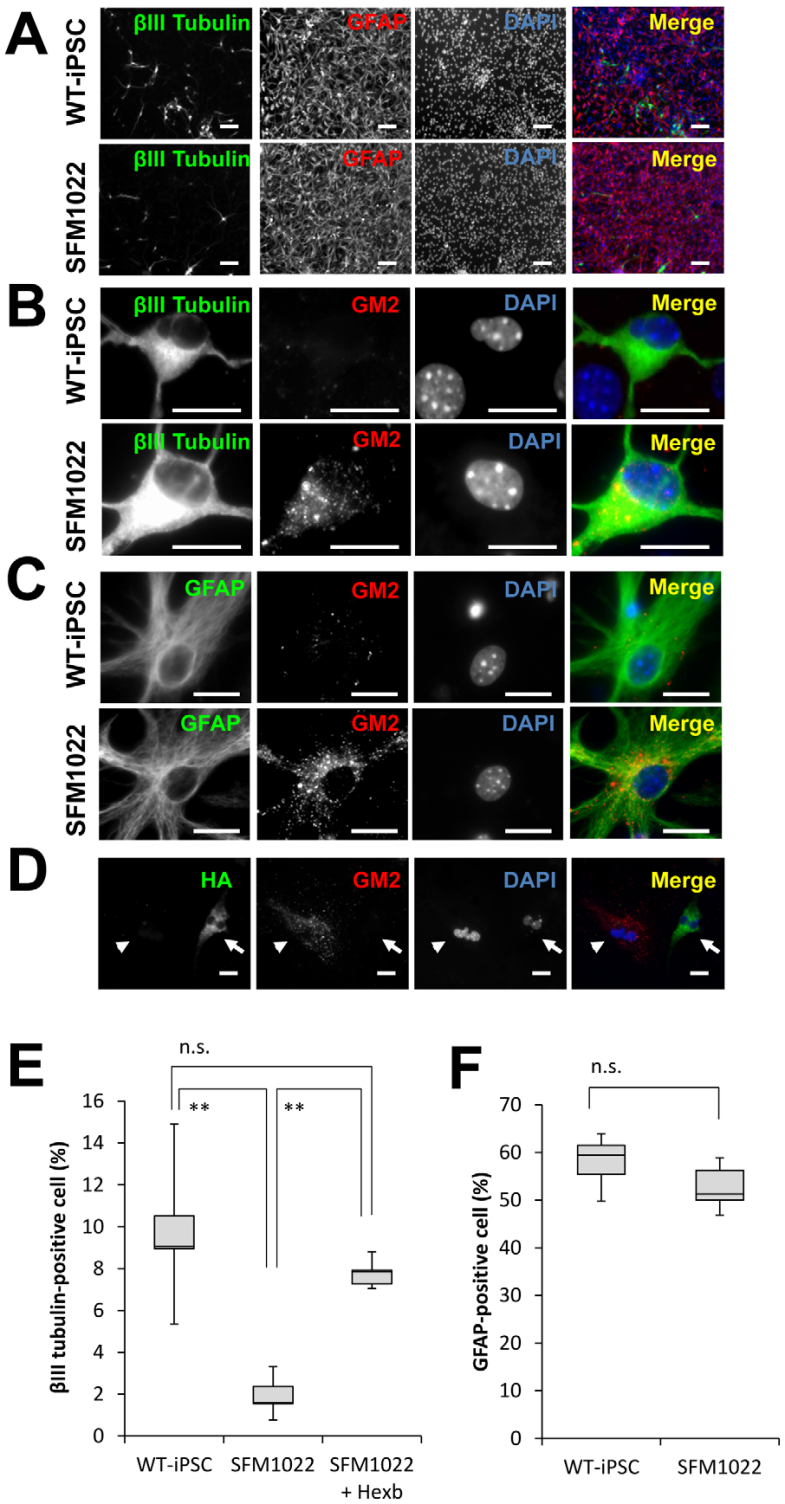
Impairment of neuronal differentiation from NSCs derived from SFM1022 cells. A–C, Immunostaining of differentiated cells for βIII tubulin (green), GFAP (green), and GM2 (red), with DAPI nuclear staining (blue). Scale bar indicates 100 µm (A) and 20 µm (B, C). D, Immunostaining of HA (green) and GM2 (red) in cells differentiated from SFM1022-derived NSCs transfected with HA-tagged Hexb cDNA. Arrows and arrowheads indicate HA-positive and -negative cells, respectively. Scale bar indicates 100 µm. E, After selection of transiently transfected cells with G418, the selected cells were allowed to differentiate toward a neural lineage. The percentages of neurons that differentiated from the NSCs of passage 1 neurospheres derived from WT-iPSCs, SFM1022, and transfected SFM1022 cells were determined. F, The percentages of astrocytes that differentiated from the NSCs of passage 1 neurospheres derived from WT-iPSCs and SFM1022 cells were determined. Data were analyzed using the Mann–Whitney U test and are shown as box-and-whisker plots. Boxes, 75^th^ percentile with the median indicated; bars, 10^th^ and 90^th^ percentiles. **P<0.01. n.s.: Difference not significant (*P*>0.05). Data were obtained for five independent experiments.

Immunostaining for GM2 ganglioside, the hallmark of SD, revealed punctuate positive signals in SD-iPSC-derived colonies (Fig. 3C), neurospheres (Fig. 4C) and differentiated neural cells (Fig. 6B, 6C). No positive signals were detected in WT-iPSCs. TLC analysis demonstrated that the content of GM2 in SFM1022 was significantly higher than that in WT-iPSCs (11 ng/1×10^6^ SFM1022 vs. 4.5 ng/1×10^6^ WT-iPSCs (Fig. 5A). Electron microscopy also showed intracytoplasmic inclusion bodies in SFM1022, but not in WT-iPSCs (Fig. 5B).

### Impaired Neuronal Differentiation of SD-iPSCs

To compare the ability of differentiation from NSCs into neural cells between SD-iPSCs and WT-iPSCs, primary neurospheres were cultured under differentiation conditions, and then immunostained to determine the relative percentages of differentiated neurons and astrocytes (Fig. 6A–6C). Although there was no difference in the ability of neurosphere formation between SD-iPSCs and WT-iPSCs, a significant reduction was observed in the ratio of neuronal differentiation in SD neurospheres, compared with that in WT neurospheres (Figs. 6E and S4A). In contrast, there was no difference in the astrocyte quantity between SD-iPSCs and WT-iPSCs (Figs. 6F and S4B). There was no apparent difference in the amount of cleaved caspase-3 between SD-iPSCs and WT-iPSCs when differentiated neurons were immunostained (Fig. S5), indicating the impaired neuronal differentiation observed in SD neurospheres was not due to their apoptosis.

To determine whether or not the impaired neuronal differentiation was due to *Hexb* gene mutation, we transfected SFM1022 cells with a plasmid encoding HA-tagged *Hexb*, and then examined the efficiency of neuronal differentiation. Immunostaining revealed that differentiated cells derived from HA-tag-positive SFM1022 cells, showed significantly decreased GM2, indicating decreased GM2 accumulation following restoration of β-hexosaminidase activity (Fig. 6D). After selection of transiently transfected cells with G418, the selected cells were allowed to differentiate toward a neural lineage. Transfection of SFM1022 cells with the *Hexb* gene reversed the impairment of neuronal differentiation (Fig. 6E).

## Discussion

In the present study, we established iPSCs from a mouse model of SD, which provides a new system to study the disease's pathogenesis during embryonic development. The initial methods to drive iPSCs used viral vectors [18]–[21], in which both the vector and transgenes are permanently integrated into chromosomes. Such vectors can produce insertional mutations that interfere with normal function, and residual transgene expression can influence differentiation into specific lineages. To avoid these disadvantages, we used an oriP/EBNA-1-based episomal vector for reprogramming. The oriP/EBNA1-based vector contributes to replication and retention of plasmids during each cell division, which is long enough for reprogramming to occur, and are lost over time, resulting in cells that are free of transfected DNA and integrated transgenes [22], [23]. The SD-iPSCs described here are pluripotent based on the similarities to murine iPSCs in terms of gene expression, proliferation and ability to spontaneously differentiate into cells of different germ layers. Notably, we have differentiated these cell lines into neural cells, one of the major cell types relevant to SD. Moreover, these cells exhibit disease-specific accumulation of GM2. These iPSCs provide an advantage to investigate the early changes caused by SD during early lineage differentiation.

The inclusion bodies observed in SD-iPSCs did not exhibit a typical membranous cytoplasmic body-like structure (Fig. 5B). There is evidence that the amount of gangliosides in the nervous system undergoes remarkable changes during development [24]. The content of GM2 is low at embryonic days 12–14 in mouse brains. The content of GM2 increases after embryonic day 16 and reaches its peak in the adult brain, which is accompanied by an increase in GM2 synthase (GalNAc transferase) expression. It is very likely that sufficient GM2 was not accumulated because of the extremely low activity of GM2 synthase in immature and undifferentiated SD-iPSCs.

In this study, we generated iPSCs from NSCs. Kim *et al*. achieved the generation of iPSCs from mouse NSCs and showed that they are efficiently transmitted through the germline [25], indicating that the iPSCs generated from NSCs are germline-competent cells similar to mouse ES cells. It is therefore conceivable that the SD-iPSCs described here have mouse iPSC-specific properties. Moreover, we observed that reprogrammed SD-iPSCs showed impaired neuronal differentiation compared with that of WT-iPSCs from the same origin of fibroblasts. Transfection of SD-iPSCs with the *Hexb* gene reversed the impairment of neuronal differentiation. Taken together, the present findings reflect the *Hexb*-null phenotype, and not the origin of the NSCs.

The *Hexb*−/− mice develop an SD-like illness and are therefore a useful animal model to investigate the pathophysiology of SD. In addition, *Hexb*−/− mice are born and grow without obvious neurological defects until 3 months of age, at which time they develop a tremor, startle reaction and increased limb tone. These manifestations become progressively more severe until 4–5 months after birth [2], [3], [26]. Postmortem examination reveals marked loss of neurons in the cerebral cortex and cerebellum, as well as extensive gliosis in SD patients [27]. Accordingly, *Hexb*−/− mice have been reported to exhibit neuronal loss in the thalamus, brainstem and spinal cord in association with apoptotic signals at the symptomatic stage (4 months of age) [4], [28]. However, it is still unknown how excess accumulation of glycolipids ultimately leads to the neurological defects. Pelled *et al.* showed normal viability but reduced rates of axonal and dendritic growth in cultured neurons from the hippocampus of embryonic *Hexb*−/− mice [29]. Sango *et al.* reported impaired survival but normal neurite outgrowth in cultured dorsal root ganglion neurons from presymptomatic *Hexb*−/− mice (4–5 weeks of age) [30]. These results suggest that morphological and functional abnormalities of neurons beginning at the embryonic and presymptomatic stages trigger the progressive neurological manifestations that occur at the later symptomatic stage.

During neural differentiation, the promoters of many genes related to the neural lineage undergo demethylation of histones, resulting in increased expression of these genes [31], [32]. The machinery of histone methylation/demethylation is regulated by cell signaling molecules such as Notch, bFGF, LIF, Shh, and Wnt [33], [34]. Several gangliosides play a pivotal role in embryonic development and neuronal differentiation [35]–[37], and regulate growth factor-mediated cell signaling pathways for neural development [38]. For example, GM3 ganglioside is a negative regulator of the epidermal growth factor receptor (EGFR) [39]–[41], a key negative regulator of Notch1 gene expression [42]. Another ganglioside, GM1, enhances neural differentiation by lipid raft-dependent activation of growth factor receptors such as the FGF-2 and LIF receptors [43], [44]. In the present study, we found impaired differentiation of SD-iPSCs into NSCs. It is quite easy to theorize that the deficient activity of HexA and HexB due to *Hexb* mutation results in the impaired metabolism of gangliosides including GM2 in SD-iPSCs. One possible explanation of the impaired differentiation is that the accumulation of gangliosides influences the histone methylation machinery *via* the regulation of cell signaling pathways, thereby preventing upregulation of the expression of key developmental genes and resulting in impairment of the differentiation of SD-iPSCs into NSCs.

During the differentiation of ES cells into NSCs, ES cells lose their pluripotency as genes related to non-neural lineages and pluripotency become inaccessible to transcription factors and are therefore silenced [45]. Conversely, the genes related to neural lineages are poised to respond to appropriate cues in ES cells but become de-repressed upon differentiation into the neural lineage [46]. In the present study, no difference was observed in the ability to maintain NSCs between SD-iPSCs and WT-iPSCs. It is likely that NSCs derived from SD-iPSCs were fully capable of receiving the appropriate cues for the maintenance of NSCs.

We observed an impaired ability of SD-iPSCs to undergo neural development. A striking finding in this study was the disease-relevant impairment of neuronal differentiation without an effect on NSC growth. It is well known that NSCs significantly decrease neuron generation with increasing passage number *in vitro* [47], [48]. In the mammalian central nervous system, neurons are generated primarily during the embryonic period, whereas most glial cells are generated after birth. It is therefore conceivable that the differentiation capacity has temporally changed in SD-iPSCs, in which the timing of differentiation into neurons is terminated early, whereas timing of differentiation into astrocytes is accelerated. It is well known that neural differentiation is regulated in a spatial/temporal manner during the developmental stage, and therefore various types of neurons and the glial cells are localized in appropriate sites at suitable times. One possibility is that the phenotype of *Hexb*−/− mice affects intrinsic developmental programs and thereby leads to neurological dysfunction.

Temporal switching of cell differentiation is controlled by Notch signaling and epigenetic changes of gene expression, which are involved in the differentiation of neurons and astrocytes [32], [49], [50]. Notch, a single transmembrane spanning protein, is expressed in undifferentiated cells. When Notch ligands such as Delta activate Notch, the intracellular domain of Notch is transferred into the nucleus, where it forms a complex with RBP-J. This complex induces Hes1 and Hes5 expression [51]–[53], which antagonize proneural genes, thereby inhibiting neurogenesis, maintaining neural progenitors, and promoting gliogenesis [54]–[56]. Moreover, the interaction between the EGFR and Notch signaling pathway described above is reported to have fundamental and selective roles in the maintenance of NSCs [57]. In this context, the accumulated gangliosides may activate Notch signaling *via* repression of the EGFR, thereby inhibiting neurogenesis.

In conclusion, we have generated iPSCs from the SD model mouse. SD-iPSCs exhibit pluripotent stem cell properties and SD phenotypes. Although SD-iPSCs could differentiate toward a neural lineage, impaired neuronal differentiation of SD-iPSCs was observed. These results suggest that this study has provided a new system to study SD pathogenesis. However, it remains to be clarified whether or not the impairment of neuronal differentiation is reliably associated with disease-specific pathologic phenomena. Accordingly, we attempted to examine whether or not a difference in the ability to differentiate into neurons can be observed in NSCs isolated from cortices from SD mouse embryos. However, we failed to obtain enough NSCs from SD mouse brains for differentiation. The problem was caused by difficulties in obtaining enough *Hexb*−/− embryos to prepare NSCs, which was due to the impaired mating behavior in *Hexb*−/− male mice. Therefore, we cannot include the additional data in this study.

## Supporting Information

Figure S1 **The cell proliferation of SD-iPSC clones.** The cell proliferation of SD-iPSC clones (SFM1022, SFM6361, and SFM6362) and WT-iPSCs were evaluated using the WST-1 reagent. The absorbances of the dye at a wavelength of 450 nm versus culture time were plotted. Values represent the mean±S.E. from four independent experiments.
(TIF)

Figure S2 **Differentiation of SFM6361 and SFM6362 into cell types of the three germ layers.** Immunostaining showed that markers for the three germ layers (α-smooth muscle actin, α-fetoprotein, and βIII tubulin) were expressed in spontaneously differentiated SFM6361 and SFM6362 cells. WT-iPSCs were used as a positive control. Scale bar indicates 100 µm.
(TIF)

Figure S3 **Impaired differentiation of SFM6361 and SFM6362 cells into NSCs.** The number (as a function of the initial cell number plated) (A) and sizes (B) of SDIA-induced colonies of larger than 100 µm at day 7 of differentiation were determined. Data were analyzed using the Mann–Whitney U test and are shown as box-and-whisker plots. Boxes, 75^th^ percentile with the median indicated; bars, 10^th^ and 90^th^ percentiles. **P<0.01. Data were obtained from five independent experiments.
(TIF)

Figure S4 **Impairment of neuronal differentiation of NSCs derived from SFM6361 and SFM6362 cells.** A, The percentages of neurons that differentiated from the NSCs of passage 1 neurospheres derived from WT-iPSCs, SFM6361 and SFM6362 cells were determined. B, The percentages of astrocytes that differentiated from the NSCs of passage 1 neurospheres derived from WT-iPSCs, SFM6361 and SFM6362 cells were determined. Data were analyzed using the Mann–Whitney U test and are shown as box-and-whisker plots. Boxes, 75^th^ percentile with the median indicated; bars, 10^t^h and 90^th^ percentiles. **P<0.01. n.s.: Difference not significant (*P*>0.05). Data were obtained from five independent experiments.
(TIF)

Figure S5 **Immunostaining of differentiated cells for βIII tubulin (green) and cleaved caspase-3 (CC3; red).** Scale bar indicates 200 µm.
(TIF)

Table S1 Primers used for PCR.
(DOCX)

Movie S1 Beating cardiomyocytes derived from WT-iPSCs.
(MP4)

Movie S2 Beating cardiomyocytes derived from SD-iPSCs.
(MP4)

## References

[1] Yamanaka S, Johnson ON, Norflus F, Boles DJ, Proia RL (1994) Structure and expression of the mouse beta-hexosaminidase genes, Hexa and Hexb. Genomics 21: 588–596. 7959736 10.1006/geno.1994.1318

[2] Sango K, Yamanaka S, Hoffmann A, Okuda Y, Grinberg A, et al (1995) Mouse models of Tay-Sachs and Sandhoff diseases differ in neurologic phenotype and ganglioside metabolism. Nat Genet 11: 170–176. 7550345 10.1038/ng1095-170

[3] Phaneuf D, Wakamatsu N, Huang JQ, Borowski A, Peterson AC, et al (1996) Dramatically different phenotypes in mouse models of human Tay-Sachs and Sandhoff diseases. Hum Mol Genet 5: 1–14. 8789434 10.1093/hmg/5.1.1

[4] Huang JQ, Trasler JM, Igdoura S, Michaud J, Hanal N, et al (1997) Apoptotic cell death in mouse models of GM2 gangliosidosis and observations on human Tay-Sachs and Sandhoff diseases. Hum Mol Genet 6: 1879–1885. 9302266 10.1093/hmg/6.11.1879

[5] Norflus F, Tifft CJ, McDonald MP, Goldstein G, Crawley JN, et al (1998) Bone marrow transplantation prolongs life span and ameliorates neurologic manifestations in Sandhoff disease mice. J Clin Invest 101: 1881–1888. 9576752 10.1172/JCI2127 PMC508774

[6] Wada R, Tifft CJ, Proia RL (2000) Microglial activation precedes acute neurodegeneration in Sandhoff disease and is suppressed by bone marrow transplantation. Proc Natl Acad Sci USA 97: 10954–10959. 11005868 10.1073/pnas.97.20.10954 PMC27130

[7] Castaneda JA, Lim MJ, Cooper JD, Pearce DA (2008) Immune system irregularities in lysosomal storage disorders. Acta Neuropathol 115: 159–174. 17924126 10.1007/s00401-007-0296-4

[8] Oishi K, Ogawa Y, Gamoh S, Uchida MK (2002) Contractile responses of smooth muscle cells differentiated from rat neural stem cells. J Physiol 540: 139–152. 11927676 10.1113/jphysiol.2001.013278 PMC2290205

[9] Kinsella TM, Nolan GP (1996) Episomal vectors rapidly and stably produce high-titer recombinant retrovirus. Hum Gene Ther 7: 1405–1413. 8844199 10.1089/hum.1996.7.12-1405

[10] Okita K, Nakagawa M, Hyenjong H, Ichisaka T, Yamanaka S (2008) Generation of mouse induced pluripotent stem cells without viral vectors. Science 322: 949–953. 18845712 10.1126/science.1164270

[11] Takahashi K, Okita K, Nakagawa M, Yamanaka S (2007) Induction of pluripotent stem cells from fibroblast cultures. Nat Protoc 2: 3081–3089. 18079707 10.1038/nprot.2007.418

[12] Kawasaki H, Mizuseki K, Nishikawa S, Kaneko S, Kuwana Y, et al (2000) Induction of midbrain dopaminergic neurons from ES cells by stromal cell-derived inducing activity. Neuron 28: 31–40. 11086981 10.1016/s0896-6273(00)00083-0

[13] Oishi K, Ito-Dufros Y (2006) Angiogenic potential of CD44+CD90+ multipotent CNS stem cells in vitro. Biochem Biophys Res Commun 349: 1065–1072. 16962069 10.1016/j.bbrc.2006.08.135

[14] Mathew R, Jia W, Sharma A, Zhao Y, Clarke LE, et al (2010) Robust activation of the human but not mouse telomerase gene during the induction of pluripotency. FASEB J 24: 2702–2715. 20354136 10.1096/fj.09-148973 PMC2909285

[15] Ahmed RP, Haider HK, Buccini S, Li L, Jiang S, et al (2011) Reprogramming of skeletal myoblasts for induction of pluripotency for tumor-free cardiomyogenesis in the infarcted heart. Circ Res 109: 60–70. 21566212 10.1161/CIRCRESAHA.110.240010 PMC3155953

[16] Tai T, Sze L, Kawashima I, Saxton RE, Irie RF (1987) Monoclonal antibody detects monosialogangliosides having a sialic acid alpha 2-3-galactosyl residue. J Biol Chem 262: 6803–6807. 3571288

[17] Silva J, Barrandon O, Nichols J, Kawaguchi J, Theunissen TW, et al (2008) Promotion of reprogramming to ground state pluripotency by signal inhibition. PLoS Biol 6: e253. 18942890 10.1371/journal.pbio.0060253 PMC2570424

[18] Takahashi K, Yamanaka S (2006) Induction of pluripotent stem cells from mouse embryonic and adult fibroblast cultures by defined factors. Cell 126: 663–676. 16904174 10.1016/j.cell.2006.07.024

[19] Wernig M, Meissner A, Foreman R, Brambrink T, Ku M, et al (2007) In vitro reprogramming of fibroblasts into a pluripotent ES-cell-like state. Nature 448: 318–324. 17554336 10.1038/nature05944

[20] Takahashi K, Tanabe K, Ohnuki M, Narita M, Ichisaka T, et al (2007) Induction of pluripotent stem cells from adult human fibroblasts by defined factors. Cell 131: 861–872. 18035408 10.1016/j.cell.2007.11.019

[21] Okita K, Ichisaka T, Yamanaka S (2007) Generation of germline-competent induced pluripotent stem cells. Nature 448: 313–317. 17554338 10.1038/nature05934

[22] Lindner SE, Sugden B (2007) The plasmid replicon of Epstein-Barr virus: mechanistic insights into efficient, licensed, extrachromosomal replication in human cells. Plasmid 58: 1–12. 17350094 10.1016/j.plasmid.2007.01.003 PMC2562867

[23] Nanbo A, Sugden A, Sugden B (2007) The coupling of synthesis and partitioning of EBV's plasmid replicon is revealed in live cells. EMBO J 26: 4252–4262. 17853891 10.1038/sj.emboj.7601853 PMC2000340

[24] Ngamukote S, Yanagisawa M, Ariga T, Ando S, Yu RK (2007) Developmental changes of glycosphingolipids and expression of glycogenes in mouse brains. J Neurochem 103: 2327–2341. 17883393 10.1111/j.1471-4159.2007.04910.x

[25] Kim JB, Zaehres H, Wu G, Gentile L, Ko K, et al (2008) Pluripotent stem cells induced from adult neural stem cells by reprogramming with two factors. Nature 454: 646–650. 18594515 10.1038/nature07061

[26] Sango K, McDonald MP, Crawley JN, Mack ML, Tifft CJ, et al (1996) Mice lacking both subunits of lysosomal beta-hexosaminidase display gangliosidosis and mucopolysaccharidosis. Nat Genet 14: 348–352. 8896570 10.1038/ng1196-348

[27] Volk BW (1964) Pathologic anatomy; Volk BW, editor. New York, USA: Grune and Stratton, Inc.

[28] Sargeant TJ, Wang S, Bradley J, Smith NJ, Raha AA, et al (2011) Adeno-associated virus-mediated expression of beta-hexosaminidase prevents neuronal loss in the Sandhoff mouse brain. Hum Mol Genet 20: 4371–4380. 21852247 10.1093/hmg/ddr364

[29] Pelled D, Riebeling C, van Echten-Deckert G, Sandhoff K, Futerman AH (2003) Reduced rates of axonal and dendritic growth in embryonic hippocampal neurones cultured from a mouse model of Sandhoff disease. Neuropathol Appl Neurobiol 29: 341–349. 12887594 10.1046/j.1365-2990.2003.00455.x

[30] Sango K, Yamanaka S, Ajiki K, Tokashiki A, Watabe K (2002) Lysosomal storage results in impaired survival but normal neurite outgrowth in dorsal root ganglion neurones from a mouse model of Sandhoff disease. Neuropathol Appl Neurobiol 28: 23–34. 11849560 10.1046/j.1365-2990.2002.00366.x

[31] Burgold T, Spreafico F, De Santa F, Totaro MG, Prosperini E, et al (2008) The histone H3 lysine 27-specific demethylase Jmjd3 is required for neural commitment. PLoS One 3: e3034. 18716661 10.1371/journal.pone.0003034 PMC2515638

[32] Hirabayashi Y, Suzki N, Tsuboi M, Endo TA, Toyoda T, et al (2009) Polycomb limits the neurogenic competence of neural precursor cells to promote astrogenic fate transition. Neuron 63: 600–613. 19755104 10.1016/j.neuron.2009.08.021

[33] Mohammad HP, Baylin SB (2010) Linking cell signaling and the epigenetic machinery. Nat Biotechnol 28: 1033–1038. 20944593 10.1038/nbt1010-1033

[34] Hsieh J, Gage FH (2004) Epigenetic control of neural stem cell fate. Curr Opin Genet Dev 14: 461–469. 15380235 10.1016/j.gde.2004.07.006

[35] Takamiya K, Yamamoto A, Furukawa K, Yamashiro S, Shin M, et al (1996) Mice with disrupted GM2/GD2 synthase gene lack complex gangliosides but exhibit only subtle defects in their nervous system. Proc Natl Acad Sci U S A 93: 10662–10667. 8855236 10.1073/pnas.93.20.10662 PMC38211

[36] Hakomori S (1990) Bifunctional role of glycosphingolipids. Modulators for transmembrane signaling and mediators for cellular interactions. J Biol Chem 265: 18713–18716. 2229037

[37] Kwak DH, Seo BB, Chang KT, Choo YK (2011) Roles of gangliosides in mouse embryogenesis and embryonic stem cell differentiation. Exp Mol Med 43: 379–388. 21654188 10.3858/emm.2011.43.7.048 PMC3158496

[38] Bieberich E (2012) It′s a lipid's world: bioactive lipid metabolism and signaling in neural stem cell differentiation. Neurochem Res 37: 1208–1229. 22246226 10.1007/s11064-011-0698-5 PMC3343224

[39] Bremer EG, Schlessinger J, Hakomori S (1986) Ganglioside-mediated modulation of cell growth. Specific effects of GM3 on tyrosine phosphorylation of the epidermal growth factor receptor. J Biol Chem 261: 2434–2440. 2418024

[40] Meuillet EJ, Mania-Farnell B, George D, Inokuchi JI, Bremer EG (2000) Modulation of EGF receptor activity by changes in the GM3 content in a human epidermoid carcinoma cell line, A431. Exp Cell Res 256: 74–82. 10739654 10.1006/excr.1999.4509

[41] Miljan EA, Meuillet EJ, Mania-Farnell B, George D, Yamamoto H, et al (2002) Interaction of the extracellular domain of the epidermal growth factor receptor with gangliosides. J Biol Chem 277: 10108–10113. 11796728 10.1074/jbc.M111669200

[42] Kolev V, Mandinova A, Guinea-Viniegra J, Hu B, Lefort K, et al (2008) EGFR signalling as a negative regulator of Notch1 gene transcription and function in proliferating keratinocytes and cancer. Nat Cell Biol 10: 902–911. 18604200 10.1038/ncb1750 PMC2747621

[43] Bieberich E (2004) Integration of glycosphingolipid metabolism and cell-fate decisions in cancer and stem cells: review and hypothesis. Glycoconj J 21: 315–327. 15514480 10.1023/B:GLYC.0000046274.35732.47

[44] Yanagisawa M, Nakamura K, Taga T (2005) Glycosphingolipid synthesis inhibitor represses cytokine-induced activation of the Ras-MAPK pathway in embryonic neural precursor cells. J Biochem 138: 285–291. 16169879 10.1093/jb/mvi129

[45] Hirabayashi Y, Gotoh Y (2010) Epigenetic control of neural precursor cell fate during development. Nat Rev Neurosci 11: 377–388. 20485363 10.1038/nrn2810

[46] Mikkelsen TS, Ku M, Jaffe DB, Issac B, Lieberman E, et al (2007) Genome-wide maps of chromatin state in pluripotent and lineage-committed cells. Nature 448: 553–560. 17603471 10.1038/nature06008 PMC2921165

[47] Qian X, Shen Q, Goderie SK, He W, Capela A, et al (2000) Timing of CNS cell generation: a programmed sequence of neuron and glial cell production from isolated murine cortical stem cells. Neuron 28: 69–80. 11086984 10.1016/s0896-6273(00)00086-6

[48] Naka H, Nakamura S, Shimazaki T, Okano H (2008) Requirement for COUP-TFI and II in the temporal specification of neural stem cells in CNS development. Nat Neurosci 11: 1014–1023. 19160499 10.1038/nn.2168

[49] Kamakura S, Oishi K, Yoshimatsu T, Nakafuku M, Masuyama N, et al (2004) Hes binding to STAT3 mediates crosstalk between Notch and JAK-STAT signalling. Nat Cell Biol 6: 547–554. 15156153 10.1038/ncb1138

[50] Yoshimatsu T, Kawaguchi D, Oishi K, Takeda K, Akira S, et al (2006) Non-cell-autonomous action of STAT3 in maintenance of neural precursor cells in the mouse neocortex. Development 133: 2553–2563. 16728475 10.1242/dev.02419

[51] Hsieh JJ, Henkel T, Salmon P, Robey E, Peterson MG, et al (1996) Truncated mammalian Notch1 activates CBF1/RBPJk-repressed genes by a mechanism resembling that of Epstein-Barr virus EBNA2. Mol Cell Biol 16: 952–959. 8622698 10.1128/mcb.16.3.952 PMC231077

[52] Schroeter EH, Kisslinger JA, Kopan R (1998) Notch-1 signalling requires ligand-induced proteolytic release of intracellular domain. Nature 393: 382–386. 9620803 10.1038/30756

[53] Jarriault S, Le Bail O, Hirsinger E, Pourquie O, Logeat F, et al (1998) Delta-1 activation of notch-1 signaling results in HES-1 transactivation. Mol Cell Biol 18: 7423–7431. 9819428 10.1128/mcb.18.12.7423 PMC109323

[54] Kageyama R, Nakanishi S (1997) Helix-loop-helix factors in growth and differentiation of the vertebrate nervous system. Curr Opin Genet Dev 7: 659–665. 9388783 10.1016/s0959-437x(97)80014-7

[55] Lee JE (1997) Basic helix-loop-helix genes in neural development. Curr Opin Neurobiol 7: 13–20. 9039799 10.1016/s0959-4388(97)80115-8

[56] Ohtsuka T, Sakamoto M, Guillemot F, Kageyama R (2001) Roles of the basic helix-loop-helix genes Hes1 and Hes5 in expansion of neural stem cells of the developing brain. J Biol Chem 276: 30467–30474. 11399758 10.1074/jbc.M102420200

[57] Aguirre A, Rubio ME, Gallo V (2010) Notch and EGFR pathway interaction regulates neural stem cell number and self-renewal. Nature 467: 323–327. 20844536 10.1038/nature09347 PMC2941915

