# Supplementary figures and images for: Impaired Neural Differentiation of Induced Pluripotent Stem Cells Generated from a Mouse Model of Sandhoff Disease
Source: PLoS One. 2013 Jan 31;8(1):e55856. doi: 10.1371/journal.pone.0055856 (PMC3561340; doi:10.1371/journal.pone.0055856)

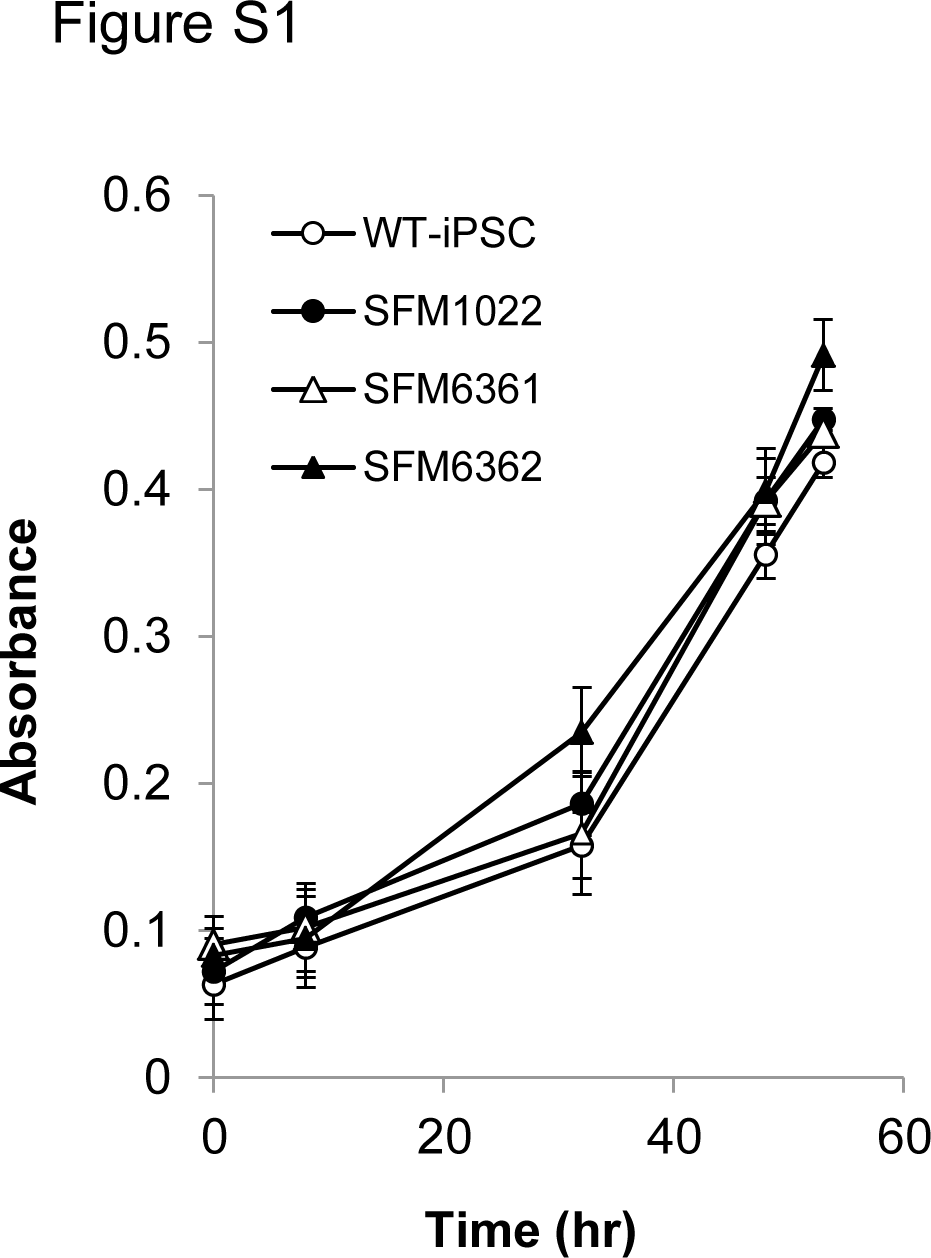

Supplement: Figure S1 — The cell proliferation of SD-iPSC clones. The cell proliferation of SD-iPSC clones (SFM1022, SFM6361, and SFM6362) and WT-iPSCs were evaluated using the WST-1 reagent. The absorbances of the dye at a wavelength of 450 nm versus culture time were plotted. Values represent the mean±S.E. from four independent experiments. (TIF) [file pone.0055856.s001.tif]

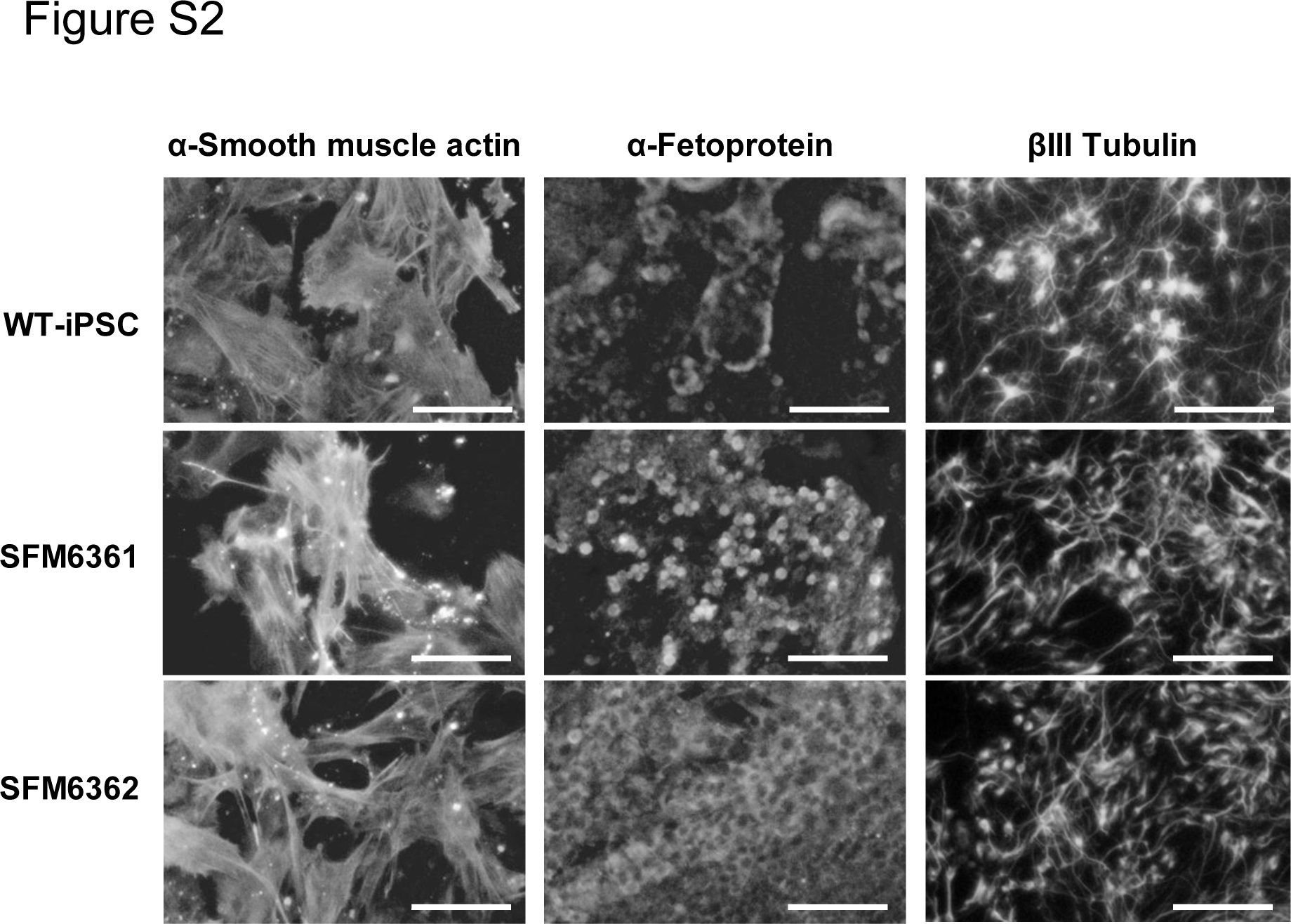

Supplement: Figure S2 — Differentiation of SFM6361 and SFM6362 into cell types of the three germ layers. Immunostaining showed that markers for the three germ layers (α-smooth muscle actin, α-fetoprotein, and βIII tubulin) were expressed in spontaneously differentiated SFM6361 and SFM6362 cells. WT-iPSCs were used as a positive control. Scale bar indicates 100 µm. (TIF) [file pone.0055856.s002.tif]

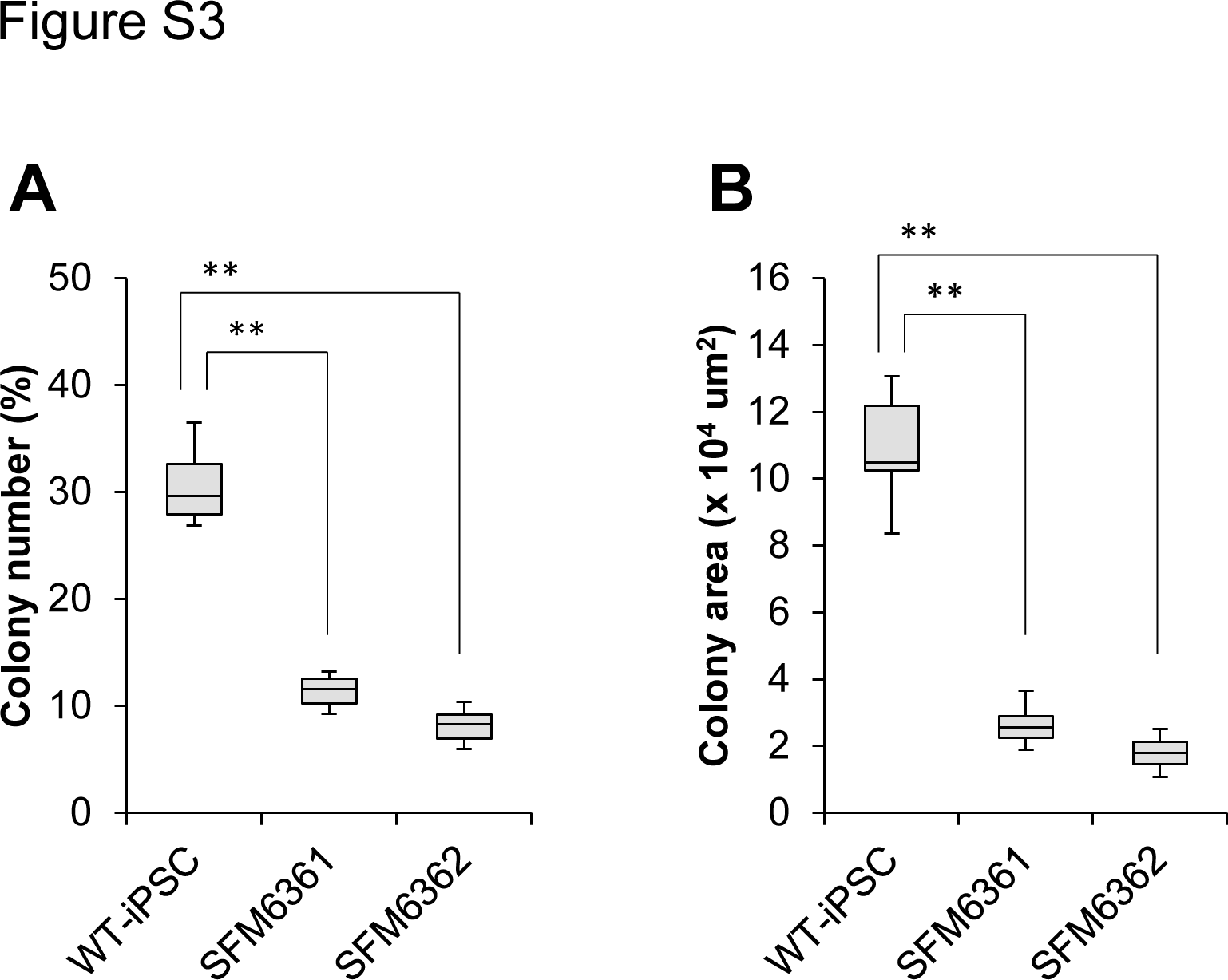

Supplement: Figure S3 — Impaired differentiation of SFM6361 and SFM6362 cells into NSCs. The number (as a function of the initial cell number plated) (A) and sizes (B) of SDIA-induced colonies of larger than 100 µm at day 7 of differentiation were determined. Data were analyzed using the Mann–Whitney U test and are shown as box-and-whisker plots. Boxes, 75th percentile with the median indicated; bars, 10th and 90th percentiles. **P<0.01. Data were obtained from five independent experiments. (TIF) [file pone.0055856.s003.tif]

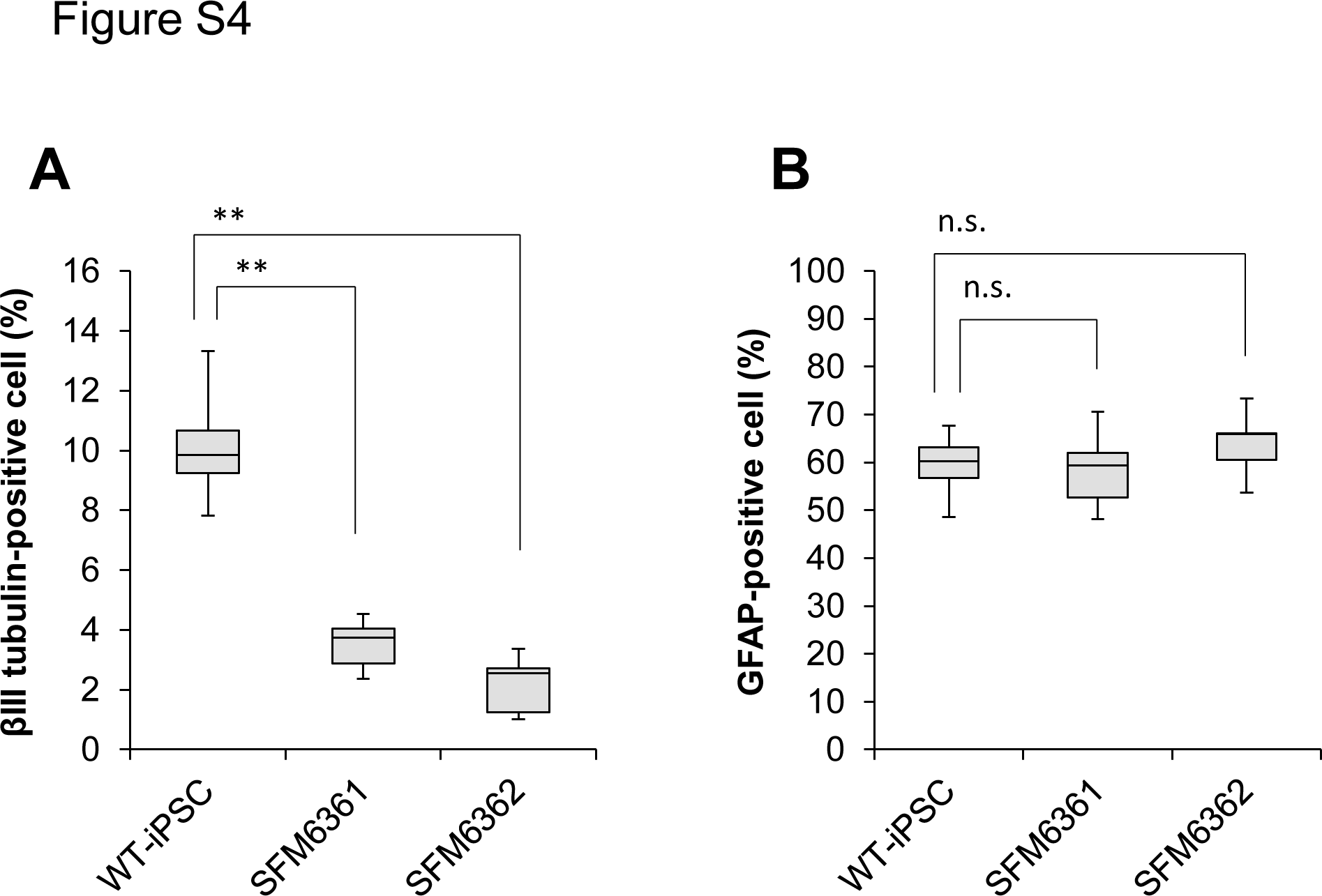

Supplement: Figure S4 — Impairment of neuronal differentiation of NSCs derived from SFM6361 and SFM6362 cells. A, The percentages of neurons that differentiated from the NSCs of passage 1 neurospheres derived from WT-iPSCs, SFM6361 and SFM6362 cells were determined. B, The percentages of astrocytes that differentiated from the NSCs of passage 1 neurospheres derived from WT-iPSCs, SFM6361 and SFM6362 cells were determined. Data were analyzed using the Mann–Whitney U test and are shown as box-and-whisker plots. Boxes, 75th percentile with the median indicated; bars, 10th and 90th percentiles. **P<0.01. n.s.: Difference not significant (P>0.05). Data were obtained from five independent experiments. (TIF) [file pone.0055856.s004.tif]

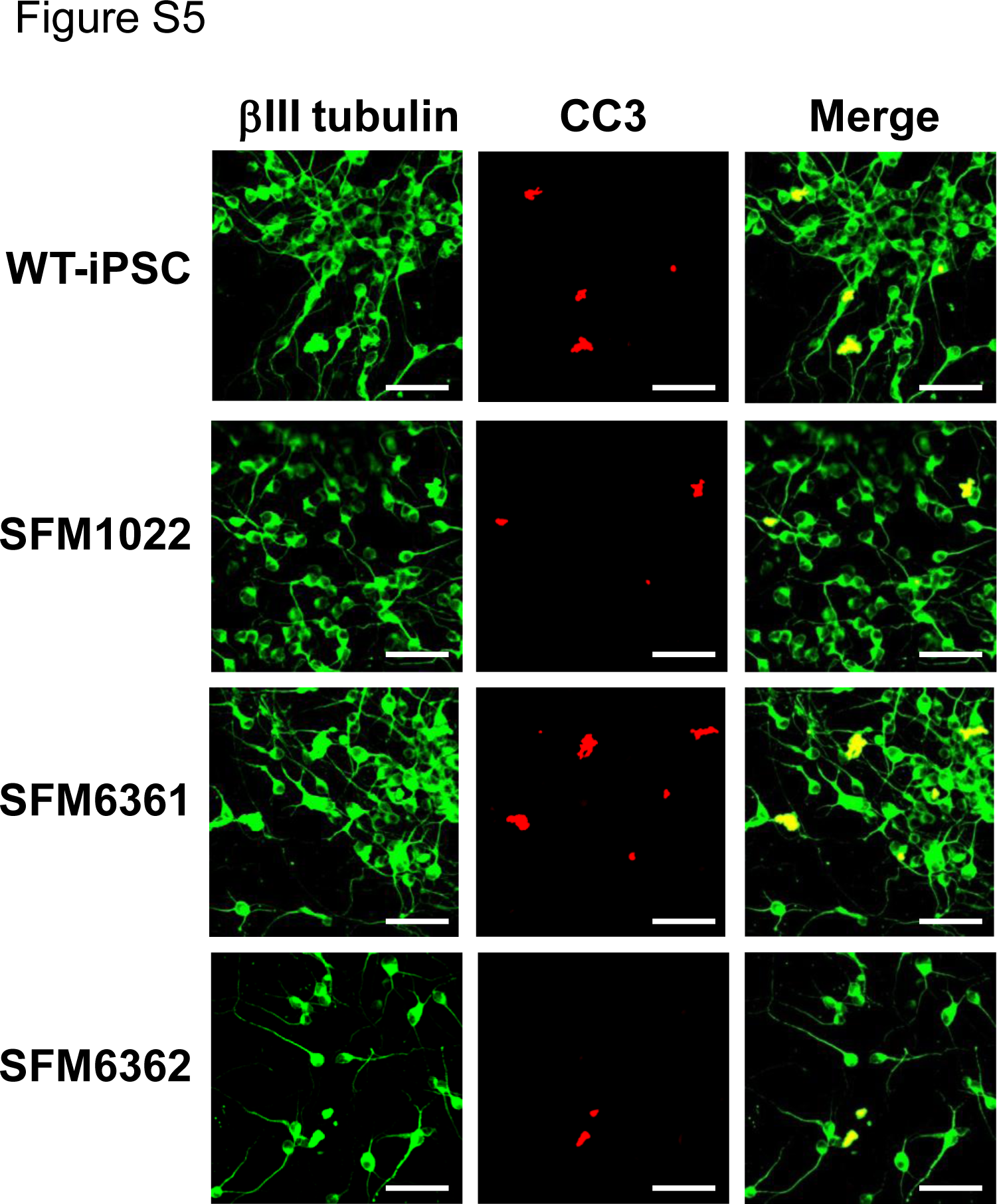

Supplement: Figure S5 — Immunostaining of differentiated cells for βIII tubulin (green) and cleaved caspase-3 (CC3; red). Scale bar indicates 200 µm. (TIF) [file pone.0055856.s005.tif]
