# Supplementary material for: Impaired Neural Differentiation of Induced Pluripotent Stem Cells Generated from a Mouse Model of Sandhoff Disease
Source: PLoS One. 2013 Jan 31;8(1):e55856. doi: 10.1371/journal.pone.0055856 (PMC3561340; doi:10.1371/journal.pone.0055856)
Supplement: Table S1 — Primers used for PCR. (DOCX) [file pone.0055856.s006.docx]

| Table S1 Primers used for PCR | | | |
| --- | --- | --- | --- |
|  |  | Sequence (5’ – 3’) | Product size  (bp) |
| Ecat1 | Sense | TGTGGGGCCCTGAAAGGCGAGCTGAGAT | 164 |
|  | Antisence | ATGGGCCGCCATACGACGACGCTCAACT |  |
| Nanog | Sense | CAGGTGTTTGAGGGTAGCTC | 223 |
|  | Antisence | CGGTTCATCATGGTACAGTC |  |
| ERas | Sense | ACTGCCCCTCATCAGACTGCTACT | 210 |
|  | Antisence | CACTGCCTTGTACTCGGGTAGCTG |  |
| Gdf3 | Sense | GTTCCAACCTGTGCCTCGCGTCTT | 570 |
|  | Antisence | AGCGAGGCATGGAGAGAGCGGAGCAG |  |
| Oct3/4 | Sense | TCTTTCCACCAGGCCCCCGGCTC | 224 |
|  | Antisence | TGCGGGCGGACATGGGGAGATCC |  |
| Sox2 | Sense | TAGAGCTAGACTCCGGGCGATGA | 297 |
|  | Antisence | TTGCCTTAAACAAGACCACGAAA |  |
| Fgf-4 | Sense | CGTGGTGAGCATCTTCGGAGTGG | 197 |
|  | Antisence | CCTTCTTGGTCCGCCCGTTCTTA |  |
| Rex1 | Sense | ACGAGTGGCAGTTTCTTCTTGGGA | 287 |
|  | Antisence | TATGACTCACTTCCAGGGGGCACT |  |
| Utf1 | Sense | GGATGTCCCGGTGACTACGTCTG | 344 |
|  | Antisence | GGCGGATCTGGTTATCGAAGGGT |  |
| Cripto | Sense | ATGGACGCAACTGTGAACATGATGTTCGCA | 174 |
|  | Antisence | CTTTGAGGTCCTGGTCCATCACGTGACCAT |  |
| Dax1 | Sense | TGCTGCGGTCCAGGCCATCAAGAG | 233 |
|  | Antisence | GGGCACTGTTCAGTTCAGCGGATC |  |
| Zfp296 | Sense | CCATTAGGGGCCATCATCGCTTTC | 307 |
|  | Antisence | CACTGCTCACTGGAGGGGGCTTGC |  |
| Nat1 | Sense | ATTCTTCGTTGTCAAGCCGCCAAAGTGGAG | 223 |
|  | Antisence | AGTTGTTTGCTGCGGAGTTGTCATCTCGTC |  |
| C-Myc | Sense | TGACCTAACTCGAGGAGGAGCTGGAATC | 170 |
|  | Antisence | AAGTTTGAGGCAGTTAAAATTATGGCTGAAGC |  |
| Klf4 | Sense | GCGAACTCACACAGGCGAGAAACC | 711 |
|  | Antisence | TCGCTTCCTCTTCCTCCGACACA |  |
